# Spatial-temporal pattern of colorectal cancer mortality in a Northeastern Brazilian State

**DOI:** 10.1371/journal.pone.0298100

**Published:** 2024-02-23

**Authors:** Alex R. Moura, Mayara E. G. Lopes, Mylena S. Dantas, Adriane D. Marques, Érika de A. C. Britto, Marcela S. Lima, Hianga F. F. Siqueira, Ana C. R. Lisboa, Fernanda V. S. Moreira, Carlos A. Lima

**Affiliations:** 1 Health Sciences Graduate Program/Federal University of Sergipe, Aracaju, Sergipe, Brazil; 2 University Hospital/EBSERH/Federal University of Sergipe, Aracaju, Sergipe, Brazil; 3 University Tiradentes, Aracaju, Sergipe, Brazil; 4 Aracaju Cancer Registry, Aracaju, Sergipe, Brazil; Augusta University, UNITED STATES

## Abstract

Colorectal cancer (CRC) is one of the most common cancer types worldwide. Its increasing mortality trends, especially in emerging countries, are a concern. The aim of this study was to analyse mortality trends and spatial patterns of CRC in the state of Sergipe, Brazil, from 1990 to 2019. Trends were calculated using data from the Online Mortality Atlas and Joinpoint Regression Program 4.8.0.1. Spatial analyses were performed using the empirical Bayesian model and Moran indices calculated by TerraView 4.2.2 between 1990 to 1999, 2000 to 2009 and 2010 to 2019. A total of 1585 deaths were recorded during the study period, with 58.42% among females. Trends were increasing and constant for both sexes and all age groups studied. The highest mean annual percent change was 6.2 {95% Confidence interval (CI) 3.4;9.0} for males aged +65 years and 4.5 (95% CI 3.2;5.8) for females aged 50–64 years. There was positive spatial autocorrelation for both sexes in all periods studied when using the Moran index for Bayesian rates. In summary, a consistent trend of increasing colorectal cancer (CRC) mortality has been observed overall. Nevertheless, an altered spatial distribution among males has emerged over the studied period.

## Introduction

Colorectal cancer (CRC) is one of the most common types of cancer for both sexes worldwide. In 2020, it was estimated that there were over 900,000 deaths from CRC globally, second only to lung cancer [1]. Considered a multifactorial disease, colorectal cancer (CRC) has various primary etiologies, encompassing environmental factors such as obesity, alcohol consumption, and red meat intake, along with lifestyle factors like sedentary behavior, and genetic factors linked to specific mutations. These factors have undergone significant changes, notably in both developed and developing countries, contributing to an upward trend in incidence and mortality rates. Nonetheless, it is observed that the implementation of appropriate screening and surveillance measures holds potential in reducing these outcomes [2–4].

In Brazil, according to estimates from the National Cancer Institute José Alencar Gomes da Silva, for the triennium 2023–2025, CRC is in the second position among the most frequent cancers for both men and women [5]. In 2020, it accounted for 8.9% of all cancer deaths, totaling more than 19,000 deaths [6].

Incidence and mortality trends vary worldwide depending on the Human Development Index (HDI), which is an indicator of a country’s socioeconomic development. In CRC, higher incident cases are reported in countries with a high HDI, whereas a higher frequency of deaths is observed in regions with a lower HDI, indicating a relatively poorer prognosis in these áreas [7].

Given the importance of CRC on the global and Brazilian scene and its strong correlation with socioeconomic and lifestyle factors, this study analyzes mortality trends and their spatial pattern over time in the state of Sergipe.

## Materials and methods

Sergipe is a state in northeastern Brazil with a population of 2,211,868 people in 2022, distributed among 75 municipalities. It is the 22nd most populous of the 27 Brazilian states and has a Human Development Index (HDI) of 0.665 [8]. To examine CRC mortality trends and spatial patterns, we utilized a time series spanning from 1990 to 2019 sourced from the Mortality Information System (SIM) provided by the State Health Department. Additionally, we cross-referenced this data with information obtained from the Online Mortality Atlas [9] to mitigate potential biases in our analysis. The variables analyzed were sex, age group divided into: 0–19 years (children and adolescents); 20–49 years (young adults); 50–64 years (middle-aged adults); over 65 years (elderly) and all ages; neoplasm topography, number of deaths, crude mortality rate, and age-standardized mortality rate.

For spatial analysis, alongside the previously mentioned data, the 2019 municipal territorial mesh for the state of Sergipe was acquired from the publicly accessible website of the Brazilian Institute of Geography and Statistics (IBGE). Notably, this data was obtained without the necessity for prior registration on the website [10]. The Coordinate Reference System (CRS) selected was the SIGARS 2000, UTM Zone 24S and EPSG code 31984. To assess the change in the geographic pattern of the aggregate of municipalities with high risk of mortality from CRC, 3 periods of 10 years were analyzed (1990 to 1999, 2000 to 2009 and 2010 to 2019).

As inclusion criteria, we considered all deaths that had as a primary or secondary cause, according to the International Classification of Diseases (ICD-10, 10th edition), the following codes: C18 (colon), C18.0 (cecum); C18.1 (vermiform appendix); C18.2 (ascending colon); C18.3 (hepatic flexure); C18.4 (transverse colon); C18.5 (splenic flexure); C18.6 (descending colon); C18.7 (sigmoid); C18.8 (colon neoplasm with invasive lesion); C18.9 (neoplasm in colon, unspecified); C19 (rectosigmoid junction), C20 (rectum). While cross-referencing data from the National Health Card (SUS—Unified Health System) and Hospital Admission Authorization (AIH) with patient records to enhance information such as parental names and occupation, inconsistencies in addresses originating from the state of Sergipe were identified. As a result, these specific cases were automatically excluded from the research.

The collected information follows the standard of the International Agency for Research on Cancer (IARC) validated in Brazil by National Cancer Institute (INCA) [11, 12].

The Research Ethics Committee of the Federal University of Sergipe approved this study with the Certificate of Presentation of Ethical Appreciation (CPEA) number 56319422.0.0000.5546 and Opinion Number: 5.329.096. All methodological phases were applied following the relevant guidelines and regulations. Patient databases were anonymized; therefore, it was not possible to obtain informed consent. Consequently, the Ethics Committee granted exemption from the requirement of informed consent, in accordance with Resolutions 466/2012 and 510/2016 of the National Health Council of Brazil.

### Statistical analysis

Descriptive, trend, and spatial distribution analyses were performed. For descriptive analysis, crude and age-standardized mortality rates per 100,000 inhabitants were calculated for each sex, with their respective confidence intervals and topographic distribution of the disease between the colon and rectum. The population of the middle period for each age group provided by the Brazilian Institute of Geography and Statistics (IBGE) [13] was used for calculating the rates. Age standardization was performed using the world standard population proposed by Segi (1960) [14] and modified by Doll (1966) [15]. The study focuses on assessing variations in mortality and incidence rates across different demographic groups, specifically by sex and age. To accomplish this, we employed Poisson regression as well as Negative Binomial regression when we encountered instances of overdispersion in the data [16].

Joinpoint Regression Program 4.8.0.1 [17] was used to calculate mortality trends, and the data were stratified into age categories. The youngest age group (0 to 19 years) was not included because mortality was zero in several years. The Joinpoint Regression Program is a statistical software that analyzes yearly standardized rates of a particular event and calculates the annual percentage change (APC) and the average annual percentage change (AAPC), determining temporal curves and identifying their inflection points (joinpoints). The program starts with the minimum number of joinpoints (e.g., 0 joinpoints, which is a straight line) and tests whether additional joinpoints are statistically significant and should be added to the model. This allows the user to test whether an apparent change in trend is statistically significant. Significance tests use the Monte Carlo permutation method [18].

For spatial analysis, QGIS 3.10.7 [19] software was used to generate maps and TerraView 4.2.2 [20] for statistical analysis. A first-order proximity matrix was created to initiate the analysis [21]. Empirical Bayesian smoothing, calculated through TerraView 4.2.2 [20], was used to smooth random fluctuations that may have occurred due to some municipalities having a small population and therefore a small number of deaths. Through this, Bayesian rates were calculated for both sexes, corrected by the multiplicative rate of 100,000. The Global Moran Index and Local Moran Index were calculated with the performance of the pseudo-significance test using 999 permutations. These indices were used with age-standardized rates and Bayesian rates.

The Moran mirroring diagram helped define critical or transition areas by comparing the mortality of each municipality with its neighbor and verifying the spatial dependence shown by the Local Index of Spatial Association (LISA) for detecting areas with significant spatial correlation [21]. The Moran Map highlights spatial patterns of possible mortality risk clusters, having neighboring municipalities with the same characteristic, being classified in the Q1 class (+/+). In the Q2(-/-) class, areas were observed in which municipalities and their neighbors have low mortality. In the Q3 (+/-) and Q4 (-/+) the municipalities were in transition risk.

## Results

### Descriptive analysis

A total of 1585 deaths from CRC were recorded by the SIM in the state of Sergipe between 1990 and 2019, of which 58.42% were female and 41.58% were male. Separating by the period studied, there were 92 deaths from colorectal cancer among men and 111 among women in the period from 1990 to 1999; 170 among men and 274 among women in the period from 2000 to 2009; and 397 deaths for men and 541 for women in the period from 2010 to 2019 (Table 1).

**Table 1:** Number of deaths from colorectal cancer separated by topography, percentage and age-standardized rate in periods (1990 to 1999, 2000 to 2009 and 2010 to 2019) in Sergipe.

**MALE**			
	**1990 to 1999**	**2000 to 2009**	**2010 to 2019**
**Number of death/ percentage (Colon)**	46 (50%)	95 (55.88%)	244 (61.66%)
**Number of death/ percentage (Rectosigmoid transition)**	10 (10.87%)	11 (6.47%)	25 (6.3%)
**Number of death/ percentage (Rectum)**	36 (39.13%)	64 (37.65%)	128 (32.24%)
**Age-standardized death rates (10 years periods)**	1.88	2.55	4.49
**FEMALE**			
	**1990 to 1999**	**2000 to 2009**	**2010 to 2019**
**Number of death/ percentage (Colon)**	62 (55.86%)	171 (62.41%)	317 (58.6%)
**Number of death/ percentage (Rectosigmoid transition)**	22 (19.82%)	13 (4.74%)	43 (7.85%)
**Number of death/ percentage (Colon)**	27 (24.32%)	90 (32.85%)	181 (32.46%)
**Age-standardized death rates (10 years periods)**	1.78	3.31	4.79

Age-standardized mortality rates (ASR) by period studied, with their respective 95% confidence intervals (CI), are described in S1 Table.

The highest standardized rate for men was 4.8 (95% CI 3.4;6.1) in 2016 and 5.9 (95% CI 4.7;7.1) for women in 2018 (S1 Table).

Table 1 represent, for males and females respectively, the number of deaths separated by cancer topography and indicate the percentage by which each topography is referred to in the periods studied.

Adapting the methods for modeling counts and rates, as described in the study, to assess differences in colorectal cancer mortality between gender categories, we employed the Poisson and Negative Binomial regression techniques outlined in Table 2. The results presented in the Table 2 indicate that the disparities observed between the categories of sex did not reach statistical significance. On the other hand, while we did observe significant differences across various age groups, it is important to note that these distinctions could be partially elucidated by the escalating incidence rates associated with advancing age.

**Table 2:** Differences between gender categories in colorrectal cancer.

	Mortality			Incidence		
	IRR	CI95%	P-Value	IRR	CI95%	P-Value
**Sex**						
**Male/Female**	1.16	0.82–1.66	0.400 ^NB^	1.12	0.80–1.57	0.500 ^NB^
**Age**						
**50-64/20-49**	8.37	7.21–9.74	<0.001 ^P^	6.19	5.61–6.83	<0.001 ^NB^
**65+/20-49**	24.0	20.9–27.6	<0.001 ^P^	15.0	13.7–16.5	<0.001 ^NB^

Subtitle: IRR–Incidence Rate Ratio. CI95%– 95% Confidence Interval. P–Poisson Regression. NB–Negative Binomial Regression.

### Trend analysis

**Fig 1:**
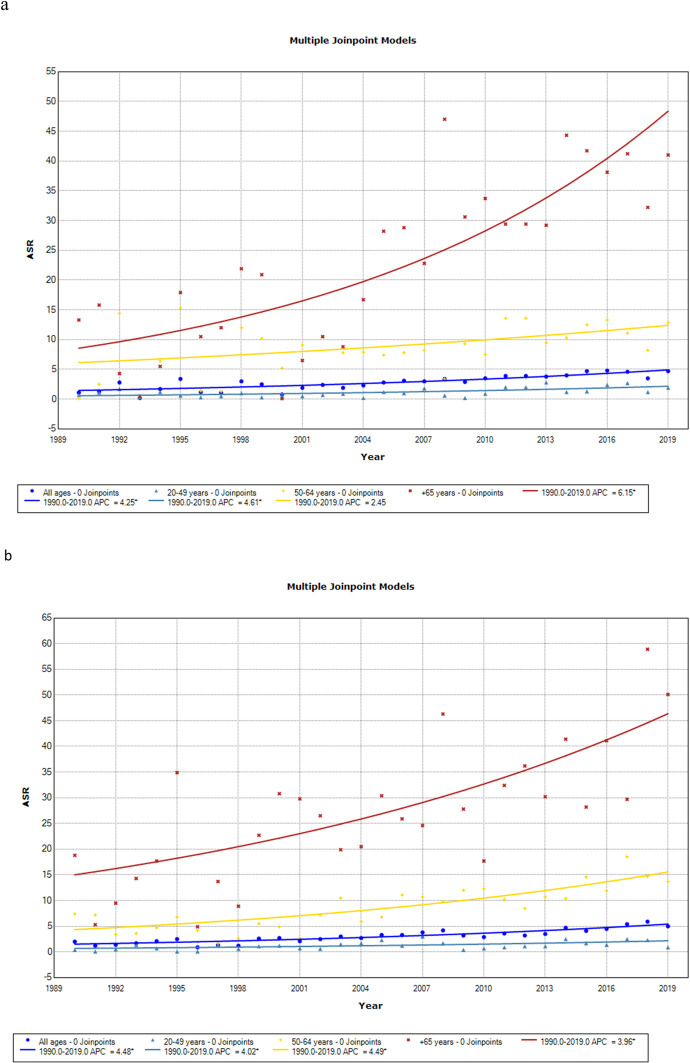
Age-standardized rates trends of colorectal cancer mortality from 1990 to 2019, according to age groups. (a) Male and (b) female.

Analyzing the mortality trends for males, as shown in Fig 1A and Table 3, a constant increase in all age groups was observed, with the highest AAPC equal to 6.2 (95% CI 3.4; 9.0) in the age group of +65 years. For females, no joinpoints were observed throughout the study period. The highest AAPC was 4.5 (95% CI 3.2; 5.8) in 50–64 years group (Fig 1B and Table 3).

**Table 3:** Joinpoint analysis for colorectal cancer mortality in males and females.

**Male**						
**Age group**	**Years**	**APC**	**95% CI**	**AAPC**	**95% CI**	**P-Value**
20–49 years	1990–2019	4.6	2.2;7.1	4.2	2.2;7.1	< 0.001
50–64 years	1990–2019	2.5	0.0;5.0	2.5	0.0;5.0	< 0.001
**+ 65 years**	1990–2019	6.2	3.4;9.0	6.2	3.4;9.0	0.05
**All ages**	1990–2019	4.2	2.9;5.6	4.2	2.9;5.6	< 0.001
**Female**						
**Age group**	**Years**	**APC**	**95% CI**	**AAPC**	**95% CI**	**P-Value**
**20–49 years**	1990–2019	4.0	1.1;7.1	4.0	1.3;7.2	< 0.001
**50–64 years**	1990–2019	4.5	3.2;5.8	4.5	3.2;5.8	< 0.001
**+ 65 years**	1990–2019	4.0	2.5;5.5	4.0	2.5;5.5	< 0.001
**All ages**	1990–2019	4.5	3.6;5.4	4.5	3.6;5.4	< 0.001

### Spatial analysis

The number of deaths was higher in the state capital, Aracaju, for both sexes, with an percentage value of 37.9%, 28.1% and 23.9% for females in the periods 1990–1999, 2000–2009, and 2010–2019, respectively. For males, 26.6%, 19.6% and 16% deaths from colorectal cancer were recorded in the same periods. Additionally, the municipalities of Nossa Senhora do Socorro, Lagarto, Itabaiana, Estância, and São Cristóvão also presented high numbers of deaths.

There was no spatial autocorrelation for standardized mortality rates by total deaths in both sexes, according to the calculation of Moran Indices (S2 Table). However, existed a positive autocorrelation for Bayesian rates for both sexes in all periods studied (S3 Table).

**Fig 2:**
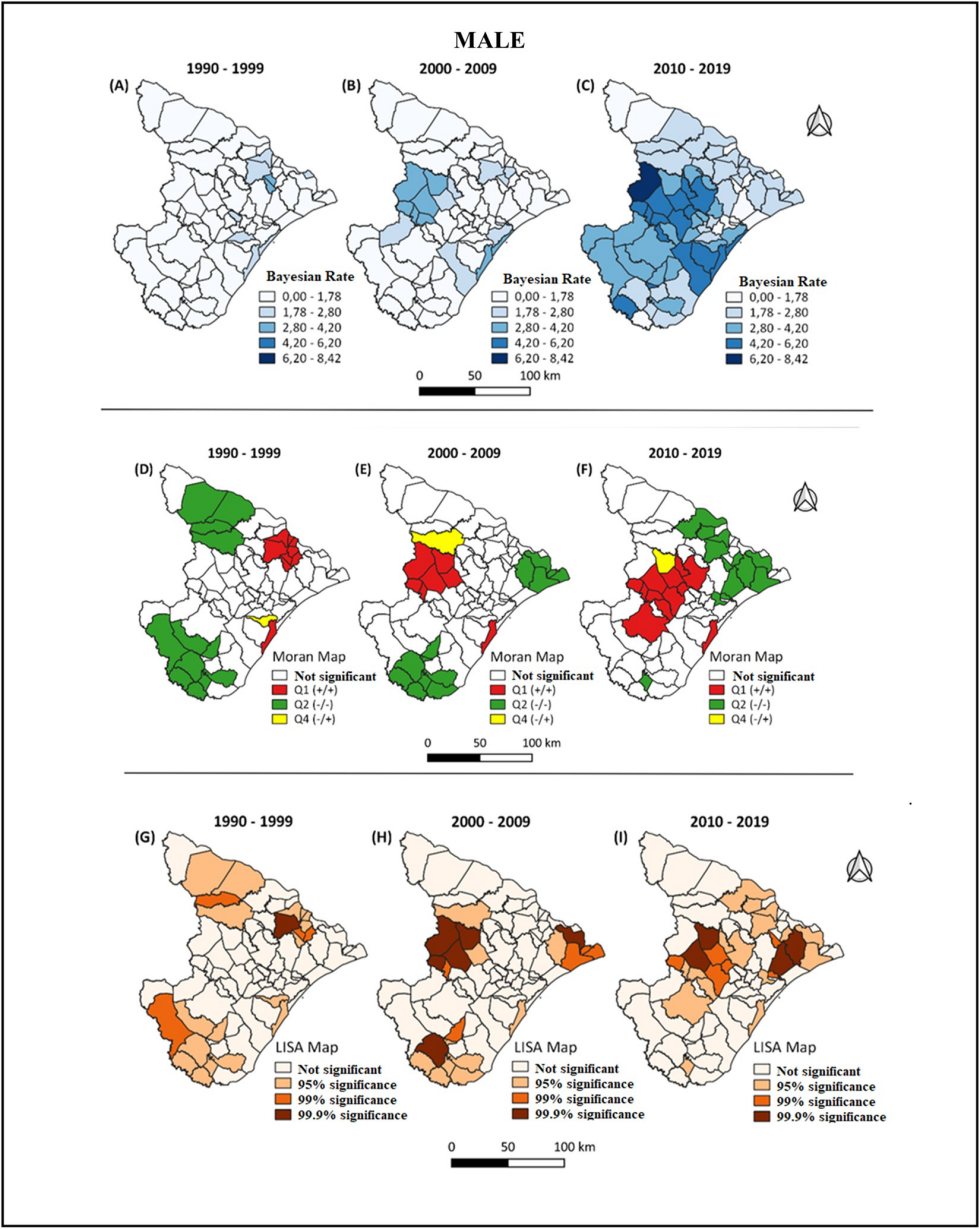
Geographical distribution of the Bayesian rate for mortality, for males, divided into 10-year intervals. Territorial grid extracted from the public domain website of IBGE (Brazilian Institute of Geography and Statistics): https://www.ibge.gov.br/geociencias/organizacao-do-territorio/malhas-territoriais.html. Source modified by the author.

**Fig 3:**
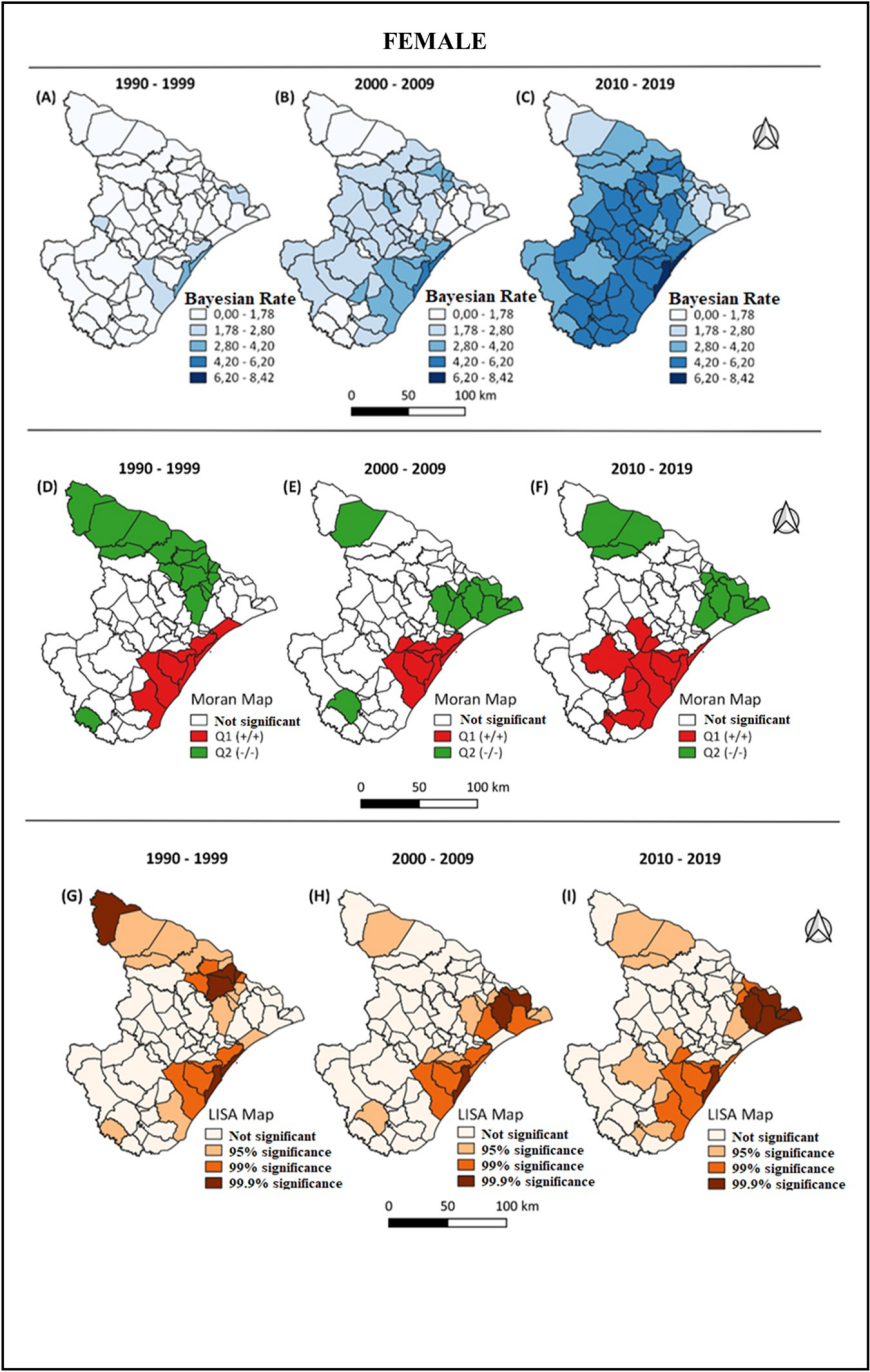
Geographical distribution of the Bayesian rate for mortality, for females, divided into 10-year intervals. Territorial grid extracted from the public domain website of IBGE (Brazilian Institute of Geography and Statistics): https://www.ibge.gov.br/geociencias/organizacao-do-territorio/malhas-territoriais.html. Source modified by the author.

To enable better visualization and analysis of spatial groupings, Moran Map and LISA Map were also constructed (Figs 2 and 3). Through these maps, it was possible to observe municipalities with high mortality rates that were likely to be close to other municipalities with high mortality rates (Figs 2 and 3), indicating areas of greater severity. At the same time, it was possible to observe municipalities with low mortality rates that were likely to be close to other municipalities with low mortality rates, indicating areas of lower severity (Figs 2 and 3).

## Discussion

The study’s findings reveal consistent and escalating trends in CRC mortality across all age groups for both men and women examined. Spatial analysis indicates that the state capital exhibits the highest risk for CRC mortality, with a positive correlation observed in both genders. Notably, among men, there has been a shift in the high-risk area over the years, with the most recent concentration of deaths observed in the central region of the state, comprising economically developed cities. Conversely, for women, the geographical cluster of elevated mortality rates has remained relatively stable in the southeastern region, where another economically developed city is situated [22, 23].

It is known that globally, the incidence and mortality rates of CRC vary according to the HDI of the studied region. The incidence tends to be higher in developed countries, with a high HDI, reflecting environmental factors and lifestyle-related to these populations [7, 24]. In contrast, the increase in mortality reflects the obstacles encountered in the health systems of less developed regions, such as delays in diagnosis and difficulty accessing treatment, so that 52% of CRC deaths occur in less developed countries [25].

Santos et al. (2019) found similar results to this study when studying trends in CRC mortality in another Brazilian state [26]. However, Siegel et al. (2017), when addressing trends in CRC mortality in the USA, found a reduction in mortality rates and pointed out early diagnosis and treatment as important factors in reducing these rates [5]. The increase in its incidence in regions experiencing accelerated development, such as Brazil, has been attributed to the increase in risk factors inherent in new habits of life acquired by the population [27, 28]. Despite this, the health system in these locations has not been growing at the same rate, which delays the diagnosis and interferes with the quality of CRC treatment, resulting in increased mortality. These results illustrate the shortcomings regarding the local public health system. In the area covered by this study, 79.3% of the population does not have private health insurance [27, 29]. Another factor that may contribute to this scenario is the absence of a screening system since CRC mortality is proven to be reduced through this action [30]. In countries with a very high HDI that have screening programs, it is already possible to notice a decrease in the incidence and mortality rates of CRC [5, 31].

In this study, the average mortality rate was higher among women compared to men, which differs from the literature. The female sex is not described as a risk factor for CRC incidence or mortality. Bray et al. (2018), in an extensive data analysis of 185 different countries, reported that age-standardized mortality rates for CRC in both sexes are similar [24]. One possible cause of this difference may be attributed to the proportion between men and women. In the 2010 census, for the state of Sergipe, the male-to-female ratio over 40 years old was 1:1.16, while in the United States, this ratio in the same period was 1:1.09 [32, 33]. In response to this scenario, regression techniques were employed, allowing for effective modeling and analysis of rates, offering valuable insights into the observed disparities among different demographic segments concerning mortality and incidence. The selected statistical approaches played a crucial role in capturing and examining the intricacies of data related to mortality and incidence variations across sex and age groups. Our analysis revealed that differences in mortality and incidence rates between sexes did not achieve statistical significance, consistent with existing literature that does not consider sex as a substantial risk factor for CRC [24]. However, concerning age-based variations, although statistically significant, the observed differences were partly influenced by the gradual increase in incidence rates associated with advancing age.

Despite evidence of a change in mortality rates, Siegel (2017) observed that the proportion of deaths for individuals under 50 years old was still the lowest among all CRC deaths, with values of 7% among men and 6% among women in 2017 in the United States [25]. In this study, as well as in Siegel’s study, it was evidenced that younger individuals presented a lower proportion among all deaths, with approximately 19% for men and 15% for women. This pattern can probably be explained by only a minority of CRC having a family origin [34]. In addition, the development of this cancer is slow, taking about 10 years to develop from an adenomatous polyp to carcinoma [35].

The most frequent site of death due to colorectal cancer (CRC) was the colon, and it was not possible to differentiate between the left or right side, as a large proportion of records were classified as "unspecified colon", reflecting the need for greater caution in recording these data by responsible professionals, as these are simple and usually easily identifiable information until the time of death. Most studies point to the left colon as the most frequent site of CRC [36, 37], but some research has shown an increase in the incidence of CRC on the right side, which changes the diagnosis and treatment of lesions, since proximal tumors have been associated with worse outcomes, with more advanced stages at the time of diagnosis and higher mortality.

Regarding the spatial distribution of CRC mortality by 10-year period, the analysis of the group of individuals by periods instead of individual risk has its epidemiological relevance. The state capital, Aracaju, was the highest-risk area for CRC mortality in all periods, with positive autocorrelation in both sexes. For males, a change in risk area was observed over the years, with the central region being the most affected region in the decade between 2010 and 2019, where there are economically developed cities such as Lagarto and Itabaiana. For females, this cluster remained relatively constant in the southeastern region of the state, where the city of Estância, another economic center, is located [8, 22]. This pattern may be related to the socioeconomic indicators of these locations, particularly the HDI [38]. However, the influence of other factors such as health investment, number of specialized centers, and screening rate should also be investigated in future studies in order to assess the importance of these factors and reproduce effective policies in localities with higher mortality rates. This method and these findings allow for the identification of geographic patterns, enabling a better understanding of the influence of local dynamics and possibly environmental and behavioral factors, providing information for better organization and strengthening of the local health system.

This study has limitations common to ecological studies, since secondary data are used, which depend on adequate filling of death certificates. However, over the years, death data has become more robust and consistent due to the better quality of death certificate completion [39]. These data are official data provided by the public Mortality Information System. Despite these limitations regarding ecological studies, the studied time series is long (1990 to 2019), presenting official data from all municipalities in the state and made it possible to observe the behavior of CRC mortality in time and space.

## Conclusion

In light of the above, it is concluded that CRC mortality in the state of Sergipe has increased in all age groups and both genders, reflecting the need for improvements in the scope of public health and questioning the need for implementation of a disease screening system in individuals over 45 years of age. In addition to this trend analysis, the evaluation of the pattern of spatial distribution of this disease allows for the determination of areas with higher and lower risk for CRC mortality, enabling the targeting of public policies to these areas with greater severity, reducing costs and contributing in the medium and long term to the reduction of its mortality.

## Supporting information

S1 Table Age-standardized rates (ASR) for male and females and confidential intervals (95% CI). (XLSX)

S2 Table Moran indices for age-standardized death rates in three periods 1990–1999; 2000–2009 and 2010–2019. (DOCX)

S3 Table Moran indices for bayesian rates in three periods 1990–1999; 2000–2009 and 2010–2019. (DOCX)

S1 File Male calculations for Sergipe mortality. (XLSX)

S2 File Female calculations for Sergipe mortality. (XLSX)

S3 File Joinpoint ASR_SE_R_Female (1990_2019). (XLSX)

S4 File Joinpoint ASR_SE_R_Male (1990_2019). (XLSX)

S5 File Sergipe CRC Mortality_Absolute values, specific rate, crude rate and standardized rate, per 100,000 women and age group, 1990–2019 year by year. (XLSX)

S6 File Sergipe CRC Mortality_Absolute values, specific rate, crude rate and standardized rate, per 100,000 men and age group, 1990–2019 year by year. (XLSX)

S7 File Female and male population and their respective standardized rates and gross death value_Colon_rectum_1990_1999. (XLSX)

S8 File Female and male population and their respective standardized rates and gross death value_Colon_rectum_2010_2019. (XLSX)

S9 File Female and male population and their respective standardized rates and gross death value_Colon_rectum_2000_2009. (XLSX)

S10 File Mortality_Incidence Ratio and their respective confidence intervals. (XLSX)
