# Supplementary material for: Spatial-temporal pattern of colorectal cancer mortality in a Northeastern Brazilian State
Source: PLoS One. 2024 Feb 23;19(2):e0298100. doi: 10.1371/journal.pone.0298100 (PMC10889879; doi:10.1371/journal.pone.0298100)
Supplement: S2 Table — (DOCX) [file pone.0298100.s002.docx]

**Table S3** **– Moran Indices for bayesian rates in three periods 1990-1999; 2000-2009 and 2010-2019.**

| 1990 – 1999 | | |
| --- | --- | --- |
| Sex | **Moran Index (I)** | **p-value** |
| Female | 0,6147 | 0,001 |
| Male | 0,4294 | 0,001 |
| 2000 – 2009 | | |
| Sex | **Moran Index (I)** | **p-value** |
| Female | 0,5582 | 0,001 |
| Male | 0,5639 | 0,001 |
| 2010 – 2019 | | |
| Sex | **Moran Index (I)** | **p-value** |
| Female | 0,5582 | 0,001 |
| Male | 0,5639 | 0,001 |
