# Supplementary material for: Spatial-temporal pattern of colorectal cancer mortality in a Northeastern Brazilian State
Source: PLoS One. 2024 Feb 23;19(2):e0298100. doi: 10.1371/journal.pone.0298100 (PMC10889879; doi:10.1371/journal.pone.0298100)
Supplement: S3 Table — (DOCX) [file pone.0298100.s003.docx]

**Table S 2** **– Moran Indices for age-standardized death rates in three periods 1990-1999; 2000-2009 and 2010-2019.**

| 1990 – 1999 | | |
| --- | --- | --- |
| Sex | **Moran Index (I)** | **p-value** |
| Female | 0,1187 | 0,078 |
| Male | -0,0605 | 0,193 |
| 2000 – 2009 | | |
| Sex | **Moran Index (I)** | **p-value** |
| Female | 0,0218 | 0,363 |
| Male | 0,1579 | 0,033 |
| 2010 – 2019 | | |
| Sex | **Moran Index (I)** | **p-value** |
| Female | 0,0226 | 0,344 |
| Male | 0,0182 | 0,401 |
